# The Great Bustard (*Otis tarda*) and Common Crane (*Grus grus*) Utilize Food Resources via Gut Microbiota Remodeling During Wintering in the Yellow River Wetlands in Ordos City, Inner Mongolia, China

**DOI:** 10.1002/ece3.73814

**Published:** 2026-06-11

**Authors:** Li Gao, Haitao Fang, Hongda Pei, Junlan Li, Li Liu

**Affiliations:** ^1^ College of Ecology and Environment Baotou Teacher's College Baotou Inner Mongolia China; ^2^ Inner Mongolia Forestry Monitoring and Planning Institute Hohhot Inner Mongolia China; ^3^ College of Life Science Inner Mongolia University Hohhot Inner Mongolia China

**Keywords:** conservation implications, dietary analysis, fecal metabolites, gut microbiota, metabolomics, wintering ecology

## Abstract

The great bustard ( *Otis tarda* ) and common crane ( *Grus grus* ), which are first‐ and second‐class protected animals in China, respectively, winter in the Yellow River wetlands of Inner Mongolia. The focus of this study was on the winter ecology of these birds in this region, in particular the relationship between their utilization of food resources and gut microbiota remodeling during the winter period. High‐throughput sequencing was used to analyze the diets and gut microbiotas of the birds, and liquid chromatography‐mass spectrometry was used to examine their fecal metabolites. The results showed that both bird species primarily consumed plant‐based food. The diet of the common crane consisted mainly of Poaceae plants, whereas that of the great bustard was relatively more diverse. The different diet components drove the diversity and composition of the gut microbiotas in the two species, with the great bustard exhibiting higher microbial diversity. The gut microbiota and metabolites of the common crane were mainly influenced by Poaceae plants, whereas those of the great bustard were influenced by multiple factors, with no single predominant driver. The gut microbiota of the great bustard contained more probiotic bacteria, whereas that of the common crane comprised a higher abundance of probiotic fungi. Moreover, the fecal metabolites of the great bustard were enriched with probiotic compounds such as flavonoids and phenolic acids, which are beneficial for overall health. This study provides insights into the survival status of these bird species, particularly the nationally protected great bustard, and offers a scientific basis for their conservation.

## Graphical abstract

This study detected the diet and gut microbiota of great bustards and common cranes in the wintering duration in the Yellow River Wetlands of Inner Mongolia using high‐throughput sequencing technology. This study indirectly indicated that great bustards and common cranes are well‐adapted to the environment of the Yellow River Wetlands during the wintering period. The wetlands provide a favorable habitat for these two rare bird species.

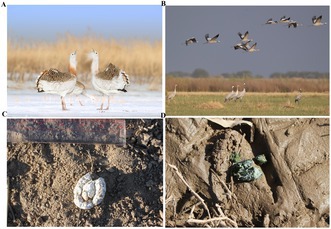

## 1 Introduction

The Yellow River wetlands of Inner Mongolia are situated within the confluence of two major migratory bird routes: the Central Asian Flyway and the East Asian–Australasian Flyway. The wetlands serve as a critical stopover and energy replenishment site for several migratory birds (Liu et al. 2019). Many waterfowl migrate to and breed in these wetlands. The great bustard ( *Otis tarda dybowskii* ; Order: Otidiformes; Family: Otididae; Genus: *Otis*), one of the largest flying bird species in the world (Martín et al. 2007), has declined markedly in numbers in recent years owing to the loss of suitable habitats due to territorial destruction, overcultivation, and overgrazing. It is currently ranked as an endangered species by the International Union for the Conservation of Nature as well as a globally vulnerable, key protected, and first‐class nationally protected bird species in China (Lu et al. 2021). Great bustards breed in high‐latitude areas, such as eastern Mongolia, Russia, and northeastern China. During winter, they migrate south to regions along the middle and lower reaches of the Yellow River in China (Kessler et al. 2013). Field surveys have shown that since around 2020, more than 10 great bustards have been observed overwintering in the Yellow River wetlands of Dalate Banner (Ordos City, Inner Mongolia) every December, and their numbers have gradually increased. The common crane ( *Grus grus* ; Order: Gruiformes; Family: Gruidae) is a second‐class nationally protected species in China. During the autumn migration period, thousands of common cranes also reach the Yellow River wetlands in Dalate Banner to replenish their energy and overwinter. The overwintering habitats of these two bird species in the Yellow River wetlands are in close proximity to each other, with similar crop types being planted in the region.

The gut microbiota refers to all microbial organisms residing in the alimentary canal of an animal host. It plays a crucial role in the host metabolism, physiology, immune function, nutrition, and disease resistance (Zhu and Xu 2025) and may even influence host behavior (Grond and Jumpponen 2018). Anthropogenic disturbances and climate change have led to a steady decline in global biodiversity. With increasing awareness of the need to conserve biodiversity, research on gut microbiotas can provide insights into the life history and underlying survival mechanisms of wildlife, which are important for informing their management and conservation. With the rapid development of high‐throughput sequencing technologies, the gut microbiotas of many bird species have been reported (Wang et al. 2026) and those of both the great bustard and the common crane have received considerable attention. Research has shown that gut microbiotas are affected by multiple factors, including the genetics, sex, and age of the host as well as its habitat and diet (Pereira et al. 2024). For example, a starch‐rich diet increases the abundance of *Lactobacillus* in the gut microbiota, whereas a diet rich in plant‐derived fibers mainly enhances *Clostridium* levels (Xiao et al. 2021). Therefore, diet influences the gut microbiota to help the host adapt to its environment and maintain its health.

Researchers have analyzed the gut bacterial diversity in great bustards in their wintering grounds in Cangzhou (Lu et al. 2021) and the composition of their diet during overwintering in the Tumuji Nature Reserve (Liu et al. 2018). However, since 2020, a small but gradually increasing number of great bustards as well as thousands of common cranes have been observed in the Yellow River wetlands of Ordos. To date, the compositions of the diets of these two bird species at this critical wintering site have not been studied nor their environmental adaptation via gut microbiota remodeling. The sympatric coexistence of these two threatened species in the Ordos wetlands represents a unique natural laboratory. The great bustard, a newly observed wintering population with a potentially broader dietary niche, and the common crane, a long‐term resident with a seemingly specialized diet (Poaceae), provide an ideal system for investigating fundamental ecological questions. Specifically, conducting an interspecific comparison allows us to (1) test whether the dietary niche breadth directly drives the gut microbial diversity and composition in wild birds under identical environmental conditions, (2) explore how different dietary regimens (diverse vs. monotonous) shape distinct gut microbial communities and their associated metabolic phenotypes, and (3) assess the adaptive strategies and physiological status of these sympatric species. To address these questions in this study, we analyzed the diets and gut microbiotas (both bacteria and fungi) of overwintering great bustards and common cranes using high‐throughput sequencing technology and profiled their fecal metabolites using liquid chromatography‐mass spectrometry (LC–MS). By integrating these datasets, we aimed to elucidate the survival status and adaptability of the two species to this critical overwintering site, thereby providing a scientific basis for their conservation, in particular that of the nationally first‐class protected great bustard in this potentially novel wintering habitat.

## 2 Materials and Methods

### 2.1 Study Area

The Dalate Banner (109°00′E–110°45′E, 40°00′N–40°30′N) terrain is high to the south and low to the north. It features hills, deserts, and tidal flats, with the southern part consisting of hilly gullies, the central part comprising the Kubuqi Desert, and the northern part covered by a Yellow River alluvial plain. The average elevation is 1080 m. The area has a temperate continental monsoon climate, with an average precipitation of 306.7 mm per year and an average temperature of 6.58°C. The area serves as a stopover site for migratory birds such as common cranes, ruddy shelducks, and swans. The sampling site is located on the southern bank of the Yellow River wetlands in Dalate Banner.

### 2.2 Sample Collection

**FIGURE 1:**
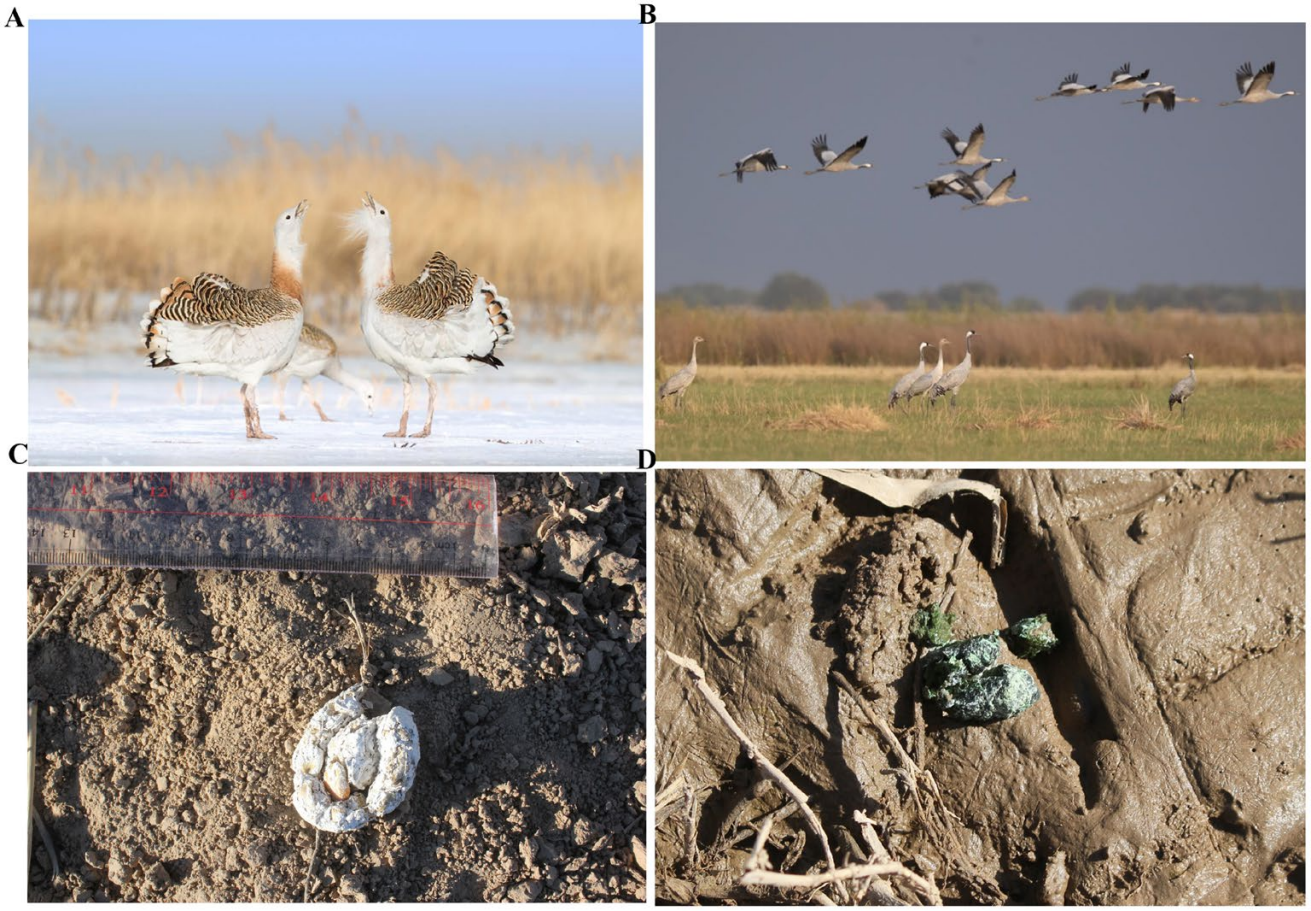
Samples. (A) The great bustards; (B) The common cranes; (C) The fecal sample of the great bustards; (D) The fecal sample of the common cranes.

Field surveys were conducted to locate the stopover sites of great bustards (Figure 1A) and common cranes (Figure 1B) by observing their activities through a monocular telescope. Because these birds rest at their stopover sites after feeding, fresh fecal samples could be collected once the birds had left the area. Fresh feces from both species (Figure 1C,D) were collected in December 2021 and stored in sterile bags. To prevent cross‐contamination, gloves were changed after each sample had been collected. To ensure that the feces were from different individuals, the samples were collected at intervals of more than 2 m. In total, five samples were collected from great bustards and six from common cranes. All samples were stored at −80°C prior to analysis.

### 2.3 Analysis of Plant‐ and Animal‐Based Diets

Total DNA was extracted from the fecal samples using the PowerSoil DNA Isolation Kit according to the manufacturer's protocol. The quality of the DNA was verified via 2% agarose gel electrophoresis. To identify the animal‐based diet components, universal primers for the cytochrome c oxidase subunit I (*COI*) gene (forward: 5′‐GCATTYCCACGAATAAATAAYATAAG‐3′; reverse: 5′‐TAAACTTCAGGGTGACCAAARAAYCA‐3′) were used to amplify animal DNA. To identify the plant‐based diet components, universal primers for the ribulose‐1,5‐bisphosphate carboxylase/oxygenase large subunit (*rbcL*) gene (forward: 5′‐CTTACCAGYCTTGATCGTTACAAAGG‐3′; reverse: 5′‐GTAAAATCAAGTCCACCRCG‐3′) were used to amplify plant DNA. The extracted DNA and primer sequences were sent to Biomarker Biotechnology Co. Ltd. (Beijing, China) for primer synthesis and sequencing. Barcode sequences, typically consisting of 6–8 nucleotides, were added to one end of each primer. The polymerase chain reaction (PCR) was performed in a final system volume of 25 μL comprising 1.5 μL of DNA template, 0.5 μL of forward primer, 0.5 μL of reverse primer, 12.5 μL of H_2_O, and 10 μL of PCR master mix. The amplicons were sequenced to obtain bulk DNA sequence data. Sequences that did not perfectly match the primers and barcodes were removed, and the primer sequences were subsequently trimmed. Sequences containing ambiguous bases (N), those with low *Q* values, and those shorter than 100 bp were excluded. The high‐quality 250 bp sequences that were retained were aligned to obtain consensus and subsequently unique sequences. Those were then clustered into operational taxonomic units (OTUs) on the basis of 97% sequence similarity. The representative OTU sequences were compared with those on the Barcode of Life Data System to obtain their taxonomic information, enabling determination of the community composition at different taxonomic levels. Species abundance tables at various taxonomic levels were generated using QIIME software. The relative abundance of a particular diet component was expressed as the percentage of its DNA sequence count relative to the total dietary DNA sequence count of the bird sample.

### 2.4 Analysis of Gut Bacteria and Fungi

Total DNA was extracted from the fecal samples using a DNA extraction kit. The V3‐V4 hypervariable regions of the bacterial 16S rRNA gene were amplified using primers 341F (5′‐CCTACGGGNGGCWGCAG‐3′) and 805R (5′‐GACTACHVGGGTATCTAATCC‐3′). The internal transcribed spacer (ITS) region of fungal DNA was amplified using primers ITS1F (5′‐ACTTGGTCATTTAGAGGAAGTAA‐3′) and ITS2R (5′‐GCTGCGTTCTTCATCGATGC‐3′). The PCR products were examined via 2% agarose gel electrophoresis and then purified, quantified, and pooled into an amplicon library for sequencing on the Illumina Novaseq 6000 platform. After quality control filtering of the sequencing data, sequences with 97% similarity were clustered into OTUs and the community composition at different taxonomic levels was analyzed. The alpha diversity of the samples was evaluated using the Shannon index. The beta diversity was analyzed using binary Jaccard‐ and Bray–Curtis‐based non‐metric multidimensional scaling analyses. The significance of differences in the beta diversity was tested using analysis of similarities (ANOSIM). Differences in bacteria and fungi between groups were analyzed using the linear discriminant analysis (LDA) effect size (LEfSe) algorithm (LDA > 4.0, *p* < 0.05).

### 2.5 Metabolite Analysis

LC–MS was used to detect the metabolites in the fecal samples. In brief, 1 g of fecal sample was placed in a 5 mL tube, mixed thoroughly with ddH_2_O, and shaken with methanol. The mixture was processed in an ultrasonic bath at ambient temperature and then centrifuged, and the supernatant was collected for concentration. The concentrated sample was dissolved in 400 μL of a methanol–water solution (methanol:water = 1:1.4) and then filtered through a 0.22 μm membrane before LC–MS analysis. MS was performed using a Waters Xevo G2‐XS QT high‐resolution mass spectrometer controlled by MassLynx V4.2 software (Liao et al. 2025). Both low‐ (2 V) and high‐collision energy (10–40 V) data were collected in dual‐channel mode, with a scanning frequency of 0.2 s per spectrum.

The raw data collected by MassLynx V4.2 were input to Progenesis QI software for peak extraction, alignment, and other data processing steps (Liao et al. 2025). Progenesis QI was used to identify the metabolites against entries in the METLIN database and a self‐built library, with theoretical fragment identification and mass error tolerance within 100 ppm. The peak areas were normalized relative to the total peak area for subsequent analysis. Principal coordinate analysis (PCoA) and Spearman's correlation analysis were used to assess sample reproducibility within groups and for sample quality control. The identified metabolites were classified and mapped to pathways using the Kyoto Encyclopedia of Genes and Genomes (KEGG), Human Metabolome, and LipidMaps databases. Differential compounds were screened on the basis of fold‐change (FC), *T*‐test *P*, and variable importance in projection (VIP) values (criteria: FC > 1, *p* < 0.05, and VIP > 1) using orthogonal partial least squares discriminant analysis (OPLS‐DA) modeling in the “ropls” R package (1.6.2) (Gao et al. 2025). In total, 200 permutation tests were performed to validate the reliability of the model.

### 2.6 Correlation Analysis

Canonical correspondence analysis (CCA) and distance‐based redundancy analysis (dbRDA) were used to analyze dietary factors with significant effects on gut microorganisms. Spearman's correlation analysis was used to assess the association between dietary factors and fecal microorganisms. CCA and Spearman's correlation analysis were also used to explore the associations between the top 10 most abundant microbial genera and top 10 differential metabolites. Heatmaps were generated to visualize the correlations. Given the inherent limitation of a small sample size when studying rare wildlife species, the correlation analyses were primarily performed for exploratory purposes. Nominal *p* values of less than 0.05 were considered as indicators of potential associations.

## 3 Results

### 3.1 Compositions of the Plant‐ and Animal‐Based Diets

High‐throughput sequencing based on the *rbcL* gene was used to analyze the plant‐based diet components of the two bird species (Table 1). The plant‐based diet of the great bustard consisted mainly of Salicaceae, Rosaceae, Amaranthaceae, Poaceae, Amaryllidaceae, unclassified_Streptophyta, Theaceae, and Fabaceae. By contrast, that of the common crane predominantly comprised Poaceae, Salicaceae, Rosaceae, and unclassified_Streptophyta species. Poaceae accounted for the largest proportion of the common crane diet and was in significantly higher abundance than that in the great bustard diet.

**TABLE 1:** Plant food composition of the two types of bird species at the family level.

Family	Relative abundance (%)	
	*Otis tarda* (OT)	*Grus grus* (GG)
Poaceae	11.6 ± 15.78	84.83 ± 13.51
Salicaceae	25.81 ± 37.7	9.69 ± 15.89
Rosaceae	21.44 ± 29.38	1.79 ± 2.53
Amaranthaceae	14.46 ± 17.65	0 ± 0
unclassified_Streptophyta	10.23 ± 9.98	1.43 ± 1.63
Amaryllidaceae	10.76 ± 21.53	0 ± 0
Fabaceae	2.41 ± 4.81	0.2 ± 0.27
Theaceae	2.42 ± 4.83	0 ± 0
Asteraceae	0 ± 0	0.42 ± 0.78
Gentianaceae	0 ± 0	0.42 ± 0.59

High‐throughput sequencing based on the *COI* gene was used to analyze the animal‐based diet composition of the two species (Table 2). For the great bustard, the primary animal‐based diet components were *Rattus*, *Arion*, unclassified_*Trichosia*, unclassified_Arthropoda, unclassified_Metazoa, and unclassified_Chordata. The primary animal components in the diet of the common crane were *Rattus*, unclassified_Metazoa, unclassified_*Ceratinella*, unclassified_Chordata, *Dasybranchus*, and *Attagenus*. Notably, *Dasybranchus* was in significantly higher abundance in the common crane diet than in the great bustard diet.

**TABLE 2:** Composition of animal diets in two bird species at the genus level.

Genus	Relative abundance (%)	
	*Otis tarda* (OT)	*Grus grus* (GG)
*Rattus*	24.75 ± 34.42	20.28 ± 11.84
Unclassified	14.77 ± 19.77	13.68 ± 12.85
unclassified_Metazoa	4.64 ± 4.79	19.63 ± 14.51
*Arion*	21.76 ± 39.01	0 ± 0
unclassified_Chordata	3.14 ± 6.28	9.22 ± 20.62
*Ceratinella*	0 ± 0	9.25 ± 20.69
*Dasybranchus*	0 ± 0	9 ± 7.09
*Trichosia*	9.16 ± 18.32	0 ± 0
unclassified_Arthropoda	8.19 ± 10.54	0.22 ± 0.35
*Attagenus*	0.11 ± 0.22	6.07 ± 8.86

### 3.2 Gut Bacteria in the Two Bird Species

The gut bacteria in the two bird species were analyzed via 16S rRNA sequencing; basic information was shown in Table 3. In total, 862,761 (98.03%) high‐quality sequences were retained after filtering of the 880,124 obtained sequences. The sequencing accuracy was high, meeting the requirements for further analysis. The 11 fecal samples yielded 376 OTUs, of which 307 were identified in the common crane samples and 352 in the great bustard samples. The average OTU count was significantly lower in the common crane samples (196.5 ± 36.37 vs. 247.6 ± 27.39; *p* < 0.05). The OTUs were classified into 14 phyla, 24 classes, 59 orders, 102 families, and 190 genera.

**TABLE 3:** Basic information of high‐throughput sequencing of 16S rRNA gene of gut bacteria in *Grus grus* (GG) and *Otis tarda* (OT).

Sample	Raw reads	Clean reads	Effective reads	Number of OTUs	Number of taxa of different taxonomic categories				
					Phylum	Class	Order	Family	Genus
GG1	80,152	79,857	79,049	231	13	22	52	81	142
GG2	79,933	79,634	78,922	140	12	17	44	68	98
GG3	79,876	79,590	78,481	198	12	21	51	78	134
GG4	79,854	79,591	78,941	179	13	21	46	70	120
GG5	80,400	80,106	79,335	191	13	21	50	77	125
GG6	79,906	79,597	78,794	240	14	23	53	87	146
OT1	80,189	79,899	78,013	236	11	19	49	77	138
OT2	79,794	79,474	77,905	233	11	18	39	65	124
OT3	80,276	79,989	78,913	286	12	21	45	81	150
OT4	79,830	79,556	76,481	218	11	19	32	57	110
OT5	79,914	79,631	77,927	265	12	19	38	64	125

**FIGURE 2:**
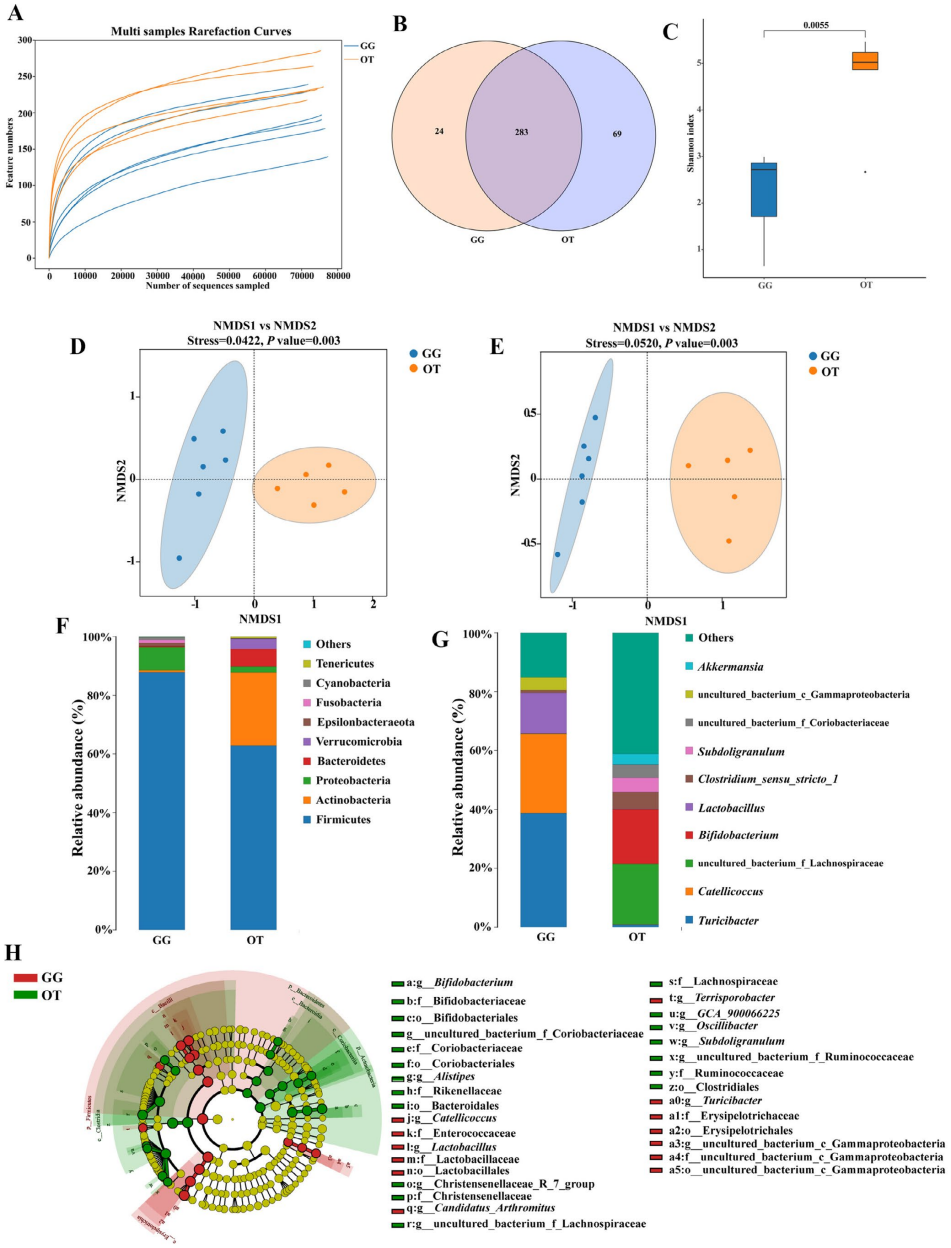
Analysis of gut bacteria in the common crane and great bustard. (A) Rarefaction curve of the gut bacteria; (B) Venn diagram of OTUs; (C) Shannon index; (D) Binary Jaccard‐based non‐metric multidimensional scaling (NMDS) analysis of bacteria; (E) Bray–Curtis‐based NMDS analysis of bacteria; (F) Bacterial phyla at greater than 1% abundance; (G) Top 10 most abundant bacterial genera; (H) LEfSe analysis.

The rarefaction curves of the gut bacteria in the 11 fecal samples flattened out (Figure 2A), indicating that the microbial diversity in the samples was adequately captured at the given sequencing depth. A Venn diagram comparing the bacterial community diversity between the two species at the OTU level revealed that 24 OTUs were unique to the common crane, 69 were unique to the great bustard, and 283 were shared between the two species (Figure 2B). At an equivalent sequencing depth, the Shannon diversity index indicated significant differences in the gut bacterial alpha diversity between the two bird species. The great bustard samples exhibited significantly higher bacterial diversity than that shown in the common crane samples (*p* < 0.05) (Figure 2C). Beta‐diversity analysis revealed the clustering of samples within the common crane and great bustard groups, with significant separation between the two (Figure 2D,E). The ANISOM results indicated significant differences in bacterial community composition between the two species (*p* < 0.05).

At the phylum level, bacterial phyla present at greater than 0.1% relative abundance included Firmicutes (75.35%), Actinobacteria (12.82%), Proteobacteria (4.93%), Bacteroidetes (3.11%), Verrucomicrobia (1.80%), Epsilonbacteraeota (0.57%), Fusobacteria (0.54%), Cyanobacteria (0.50%), and Tenericutes (0.30%). In the common crane group, the phyla at greater than 1% relative abundance were Firmicutes (87.82%), Proteobacteria (7.83%), Epsilonbacteraeota (1.13%), and Fusobacteria (1.04%). In the great bustard group, the phyla at greater than 1% relative abundance were Firmicutes (62.88%), Actinobacteria (24.89%), Proteobacteria (2.03%), Bacteroidetes (5.93%), and Verrucomicrobia (3.58%) (Figure 2F).

At the genus level, the top 10 bacterial genera at the highest abundance were *Turicibacter* (19.76%), *Catellicoccus* (13.49%), uncultured_bacterium_f_Lachnospiraceae (10.32%), *Bifidobacterium* (9.29%), *Lactobacillus* (6.91%), *Clostridium_sensu_stricto_1* (3.38%), *Subdoligranulum* (2.40%), uncultured_bacterium_f_Coriobacteriaceae (2.28%), uncultured_bacterium_c_Gammaproteobacteria (2.20%), and *Akkermansia* (1.80%). In the common crane group, the genera at greater than 1% relative abundance were *Turicibacter* (38.67%), *Catellicoccus* (26.89%), *Lactobacillus* (13.71%), and uncultured_bacterium_c_Gammaproteobacteria (4.38%). In the great bustard group, the genera at greater than 1% relative abundance were uncultured_bacterium_f_Lachnospiraceae (20.51%), *Bifidobacterium* (18.47%), *Clostridium_sensu_stricto_1* (5.839%), *Subdoligranulum* (4.80%), uncultured_bacterium_f_Coriobacteriaceae (4.56%), and *Akkermansia* (3.58%) (Figure 2G).

LEfSe analysis identified significant differences in bacterial taxa between the two bird species. The abundances of *Bifidobacterium*, uncultured_bacterium_f_Coriobacteriaceae, *Alistipes*, uncultured_bacterium_f_Lachnospiraceae, GCA_900066225, *Oscillibacter*, *Subdoligranulum*, and uncultured_bacterium_f_Ruminococcaceae were significantly higher in the great bustard group. Conversely, *Catellicoccus*, *Lactobacillus*, *Candidatus*_Arthromitus, *Terrisporobacter*, *Turicibacter*, and uncultured_bacterium_c_Gammaproteobacteria were significantly more abundant in the common crane group (Figure 2H).

### 3.3 Gut Fungi in the Two Bird Species

The gut fungi present in the common crane and great bustard were analyzed via amplicon sequencing of the ITS1 region; basic information was shown in Table 4. In total, 770,046 (95.90%) high‐quality sequences were retained after filtering of the 802,937 obtained sequences. The sequencing accuracy was high, meeting the requirements for further analysis. Of the 1136 OTUs yielded from the 11 fecal samples, 967 were identified in the common crane samples and 995 in the great bustard samples. The average OTU count was lower in the common crane samples (409.17 ± 172.04 vs. 582.00 ± 59.68), albeit the difference was not significant (*p* > 0.05). The OTUs were classified into 12 phyla, 43 classes, 92 orders, 188 families, and 371 genera.

**TABLE 4:** Basic information of high‐throughput sequencing of ITS gene of gut fungi in *Grus grus* (GG) and *Otis tarda* (OT).

Sample	Raw reads	Clean reads	Effective reads	Number of OTUs	Number of taxa of different taxonomic categories				
					Phylum	Class	Order	Family	Genus
GG1	79,751	77,682	77,169	290	8	25	54	96	152
GG2	73,773	71,354	70,576	477	9	32	67	125	227
GG3	79,898	77,689	76,990	398	10	30	60	113	192
GG4	80,148	77,993	76,317	563	11	36	77	155	283
GG5	80,131	77,418	76,630	269	8	27	51	92	146
GG6	80,047	78,226	77,956	220	10	26	54	87	131
OT1	42,277	40,959	40,285	261	9	32	66	126	209
OT2	80,042	77,797	76,476	638	11	30	70	138	235
OT3	79,892	77,489	76,564	257	9	33	72	143	249
OT4	46,951	45,371	44,690	264	10	33	72	141	251
OT5	80,027	77,403	76,393	236	9	31	73	149	275

**FIGURE 3:**
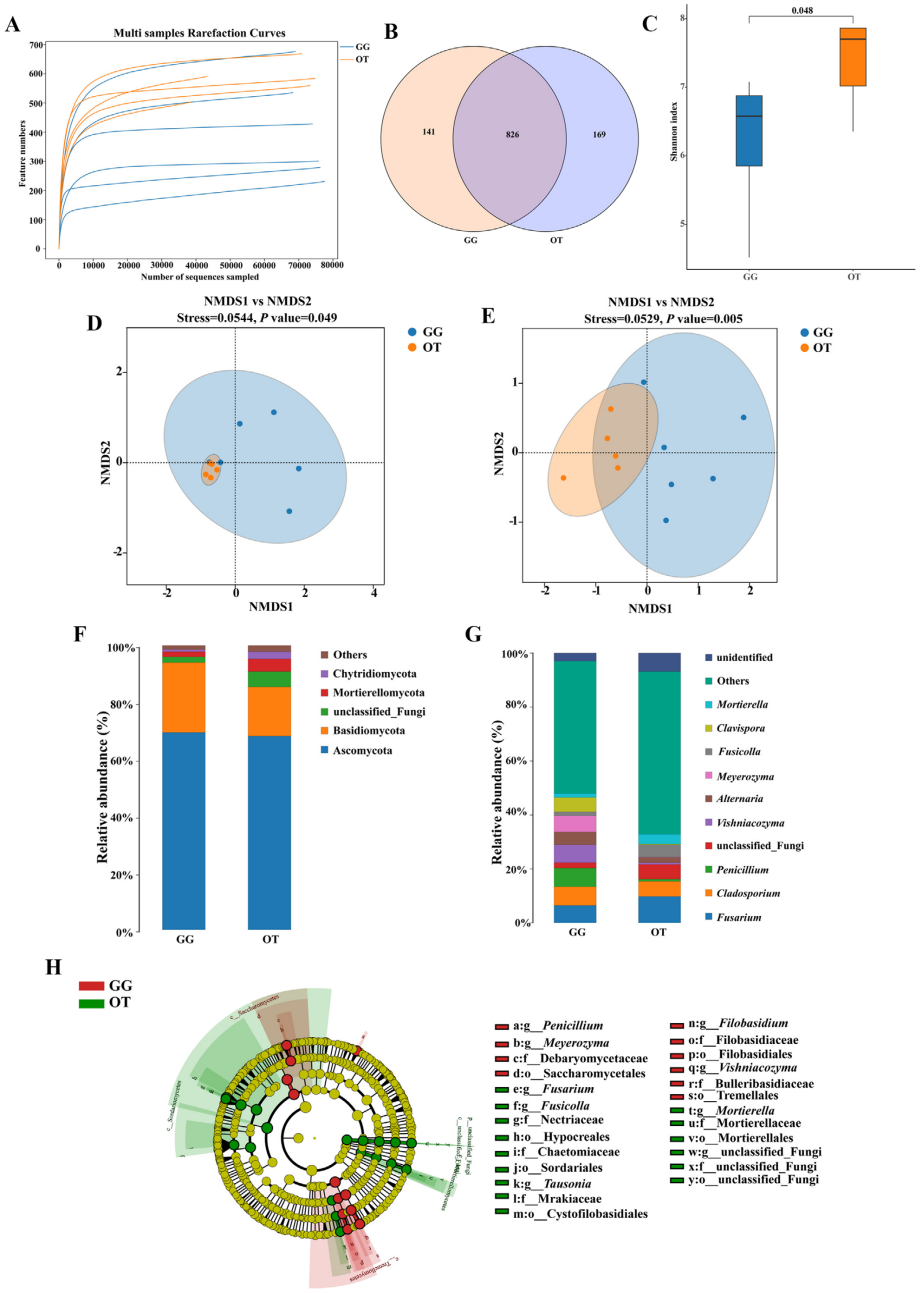
Analysis of gut fungi in the common crane and great bustard. (A) Rarefaction curve of the gut fungi; (B) Venn diagram of OTUs; (C) Shannon index; (D) Binary Jaccard‐based non‐metric multidimensional scaling (NMDS) analysis of fungi; (E) Bray–Curtis‐based NMDS analysis of fungi; (F) Fungal phyla at greater than 1% abundance; (G) Top 10 most abundant fungal genera; (H) LEfSe analysis.

The rarefaction curves of the gut fungi in the 11 fecal samples flattened out (Figure 3A), indicating that the fungal diversity in the samples was adequately captured at the given sequencing depth. A Venn diagram comparing the fungal community diversity between the two species at the OTU level revealed that 141 OTUs were unique to the common crane, 169 were unique to the great bustard, and 826 were shared between the two species (Figure 3B). At an equivalent sequencing depth, the Shannon diversity index showed significant differences in the gut fungal alpha diversity between the two bird species. The great bustard samples exhibited significantly higher fungal diversity than that shown in the common crane samples (*p* < 0.05) (Figure 3C). Beta‐diversity analysis of the fungal communities revealed clustering trends within the common crane and great bustard groups and significant separation between the two. The ANOSIM results indicated significant differences (*p* < 0.05) in fungal composition between the two bird species (Figure 3D,E).

At the phylum level, the fungi present at higher than 0.1% relative abundance were Ascomycota (68.74%), Basidiomycota (20.92%), unclassified_Fungi (3.71%), Mortierellomycota (3.12%), and Chytridiomycota (1.66%). In the common crane group, the fungal phyla at higher than 1% relative abundance were Ascomycota (68.12%), Basidiomycota (17.27%), unclassified_Fungi (5.41%), Mortierellomycota (4.43%), and Chytridiomycota (2.52%). In the great bustard group, the predominant phyla were Ascomycota (69.35%), Basidiomycota (24.57%), unclassified_Fungi (2.00%), and Mortierellomycota (1.82%) (Figure 3F).

At the genus level, the top 10 most abundant fungal genera were *Fusarium* (8.10%), *Cladosporium* (6.25%), *Penicillium* (3.91%), unclassified_Fungi (3.71%), *Vishniacozyma* (3.66%), *Alternaria* (3.35%), *Meyerozyma* (3.07%), *Fusicolla* (2.99%), *Clavispora* (2.80%), and *Mortierella* (2.53%), whereas other genera accounted for 54.72% and unidentified fungi accounted for 4.91% (Figure 3G). LEfSe analysis revealed significant differences in gut fungal composition between the two bird groups, with *Penicillium*, *Meyerozyma*, *Filobasidium*, and *Vishniacozyma* being significantly more abundant in the common crane samples and *Fusarium*, *Fusicolla*, *Tausonia*, *Mortierella*, and unclassified fungi being significantly more abundant in the great bustard samples (Figure 3H).

### 3.4 Gut Metabolites in the Two Bird Species

**FIGURE 4:**
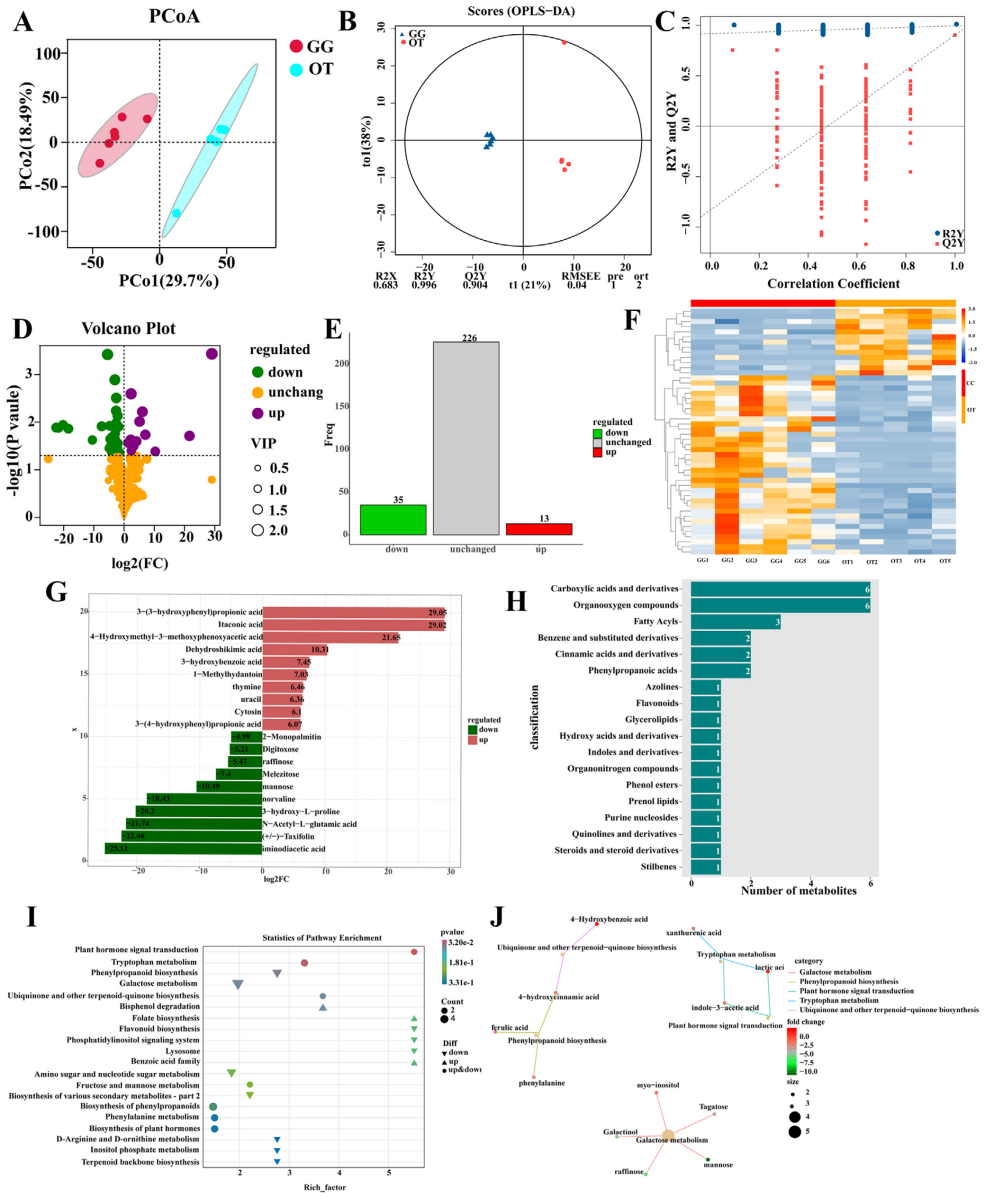
Analysis of gut metabolites in the common crane and great bustard. (A) PCoA score plot of metabolites; (B) OPLS‐DA for multivariate statistical analysis; (C) Permutation diagram with loads ranging from −1 to 1. A load close to −1 or 1 indicates a very strong effect of the variable on the component, whereas a load close to 0 indicates a very weak effect; (D) Volcano diagram for variable importance in projection (VIP) and *p*‐value screening; (E) Histogram of differential gene expression statistics, with the horizontal axis representing the individual comparison groups; (F) Heatmap of differential metabolites; (G) Top 20 fold‐change (FC) metabolites between *Grus grus* (GG) and *Otis tarda* (OT); (H) Top 20 differential metabolite classifications; (I) Top 20 KEGG pathways based on differential metabolite scores; (J) Cnetplot of enriched KEGG pathways.

PCoA of the gut metabolites (Figure 4A) showed clustering of the samples within the great bustard and common crane groups, suggesting good reproducibility within the groups and significant differences between them. PCo1 and PCo2 accounted for 29.7% and 18.49% of the total variation, respectively. The results demonstrated a significant segregating trend between the two bird species, with OPLS‐DA model parameters (R2Y = 0.996 and Q2Y = 0.904) indicating that the model was valid (Figure 4B,C). These findings suggest significant differences in gut metabolites between great bustards and common cranes.

Metabolites with a FC value of 1 or higher and a VIP value of 1 or higher were identified as differential metabolites between the two bird species. Of the 274 metabolites detected, 48 were significantly different between the two groups. Of those 48 metabolites, 35 were upregulated and 13 were downregulated in the great bustard samples (Figure 4D,E). A hierarchical clustering heatmap of the differential metabolites revealed significant differences between the two bird groups (Figure 4F). The top 10 significantly upregulated and downregulated metabolites were identified on the basis of the VIP values (Figure 4G). In the great bustard group, the top 10 significantly upregulated metabolites were 3‐(3‐hydroxyphenyl)propionic acid, itaconic acid, 4‐hydroxymethyl‐3‐methoxyphenoxyacetic acid, dehydroshikimic acid, 3‐hydroxybenzoic acid, 1‐methylhydantoin, thymine, uracil, cytosine, and 3‐(4‐hydroxyphenyl)propionic acid. The top 10 significantly downregulated metabolites in this bird group were iminodiacetic acid, (+/−)‐taxifolin, *N*‐acetyl‐l‐glutamic acid, 3‐hydroxy‐l‐proline, norvaline, mannose, melezitose, raffinose, digitoxose, and 2‐monopalmitin.

Classification and functional enrichment analyses of the differential metabolites revealed the top 20 categories to include carboxylic acids and derivatives, organooxygen compounds, fatty acyls, benzene and substituted derivatives, cinnamic acids and derivatives, phenylpropanoic acids, azolines, flavonoids, glycerolipids, hydroxy acids and derivatives, indoles and derivatives, organonitrogen compounds, phenol esters, prenol lipids, purine nucleosides, quinolines and derivatives, steroids and derivatives, and stilbenes (Figure 4H). KEGG pathway enrichment of the differential metabolites indicated that the top 20 main pathways were those of plant hormone signal transduction, tryptophan metabolism, phenylpropanoid biosynthesis, galactose metabolism, ubiquinone and other terpenoid‐quinone biosynthesis, bisphenol degradation, folate biosynthesis, flavonoid biosynthesis, phosphatidylinositol signaling system, lysosome, benzoic acid family, amino sugar and nucleotide sugar metabolism, fructose and mannose metabolism, biosynthesis of various secondary metabolites—part 2, biosynthesis of phenylpropanoids, phenylalanine metabolism, biosynthesis of plant hormones, d‐arginine and d‐ornithine metabolism, inositol phosphate metabolism, and terpenoid backbone biosynthesis (Figure 4I). Key differential metabolites that were enriched included myo‐inositol, tagatose, galactinol, mannose, and raffinose in the galactose metabolism pathway; 4‐hydroxycinnamic acid, ferulic acid, and phenylalanine in the phenylpropanoid biosynthesis pathway; lactic acid and indole‐3‐acetic acid in the plant hormone signal transduction pathway; lactic acid, indole‐3‐acetic acid, and xanthurenic acid in the tryptophan metabolism pathway; and 4‐hydroxybenzoic acid and 4‐hydroxycinnamic acid in the ubiquinone and other terpenoid‐quinone biosynthesis pathway (Figure 4J).

### 3.5 Relationships Between Dietary Factors and the Gut Microbiota in the Two Bird Species

**FIGURE 5:**
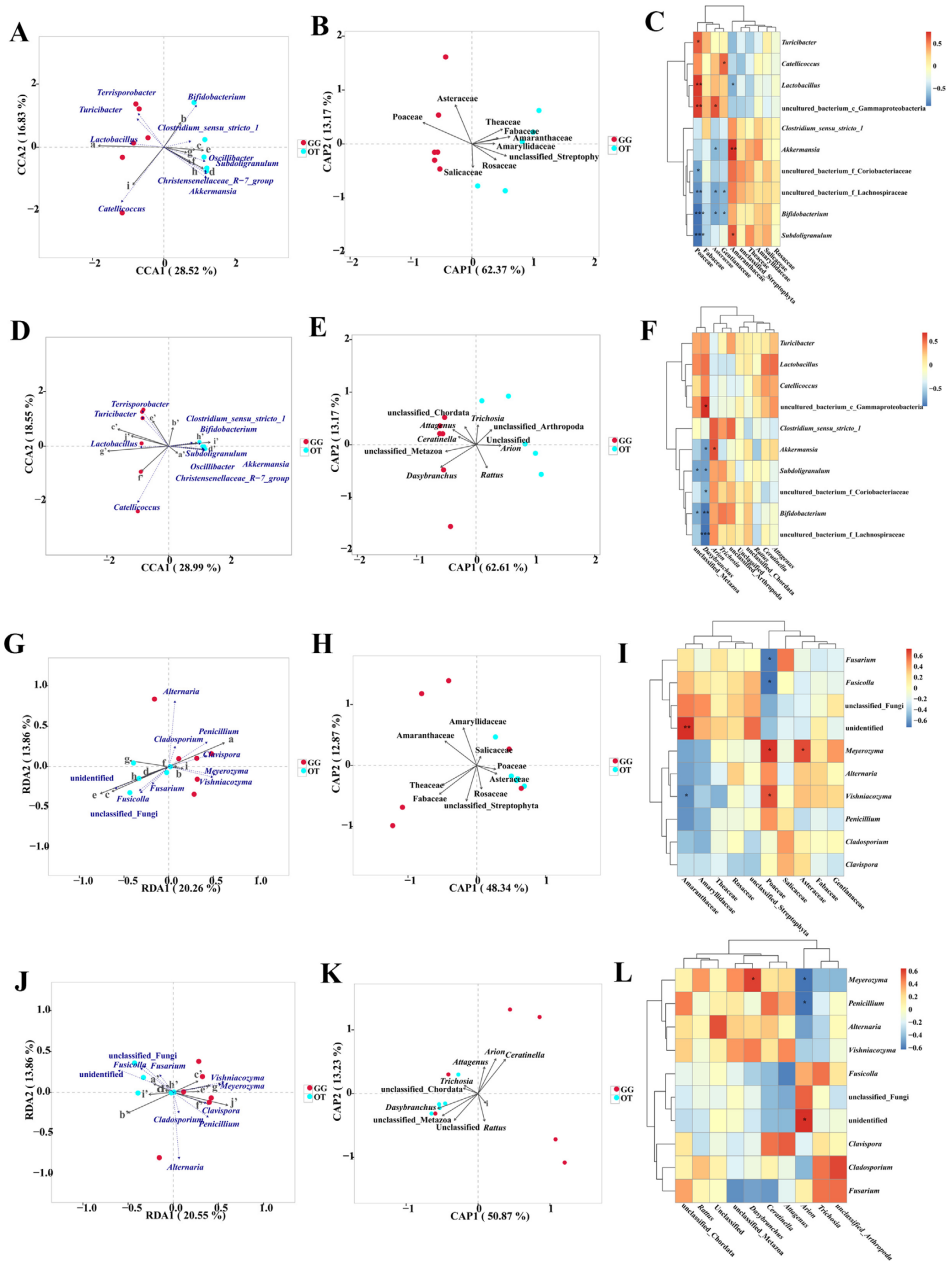
Correlations between dietary factors and the gut microbiota in the common crane and great bustard. (A) Distance‐based redundancy analysis (dbRDA)/canonical correspondence analysis (CCA) between plant‐based dietary factors and bacterial genera; (B) dbRDA between plant‐based dietary factors and bacterial genera; (C) Correlation analysis between plant‐based dietary factors and bacterial genera; (D) RDA/CCA between animal‐based dietary factors and bacterial genera; (E) dbRDA between animal‐based dietary factors and bacterial genera; (F) Correlation analysis between animal‐based dietary factors and bacterial genera; (G) RDA/CCA between plant‐based dietary factors and fungal genera; (H) dbRDA between plant‐based dietary factors and fungal genera; (I) Correlation analysis between plant‐based dietary factors and fungal genera; (J) RDA/CCA between animal‐based dietary factors and fungal genera; (K) dbRDA between animal‐based dietary factors and fungal genera; (L) Correlation analysis between animal‐based dietary factors and fungal genera. a: Poaceae; b: Salicaceae; c: Rosaceae; d: Amaranthaceae; e: Unclassified_Streptophyta; f: Amaryllidaceae; g: Fabaceae; h: Theaceae; i: Asteraceae. a’: *Rattus*; b: Unclassified; c′: unclassified_Metazoa; d′: *Arion*; e′: Unclassified_Chordata; f′: *Ceratinella*; g′: *Dasybranchus*; h′: *Trichosia*; i′: Unclassified_Arthropoda; j′: *Attagenus*.

The CCA and dbRDA results revealed the influence of food components on the bacterial communities (genus level) in the different sample groups (Figure 5). Plant‐ and animal‐based foods accounted for 45.35% and 47.54% of the total explanation rate for the gut bacterial community, respectively (Figure 5). The dbRDA results indicated that plant‐ and animal‐based foods explained 75.54% and 75.78% of the gut bacterial communities, respectively.

The plant and animal dietary factors that significantly affected the bacterial community structure in the common crane samples were Poaceae (*R* ^2^ = 0.83, *p* = 0.004) and *Dasybranchus* (*R* ^2^ = 0.44, *p* = 0.115), respectively, whereas those affecting the great bustard samples were Amaranthaceae (*R* ^2^ = 0.40, *p* = 0.087) and unclassified_Arthropoda (*R* ^2^ = 0.21, *p* = 0.43), respectively (Figure 5A,B,D,E). None of the factors affecting the great bustard samples showed statistical significance. The distinct responses of bacterial genera in the different bird samples to dietary factor levels were visualized using Spearman correlation heatmaps (Figure 5C,F). Among the plant dietary factors, Poaceae was significantly positively correlated with uncultured_bacterium_c_Gammaproteobacteria, *Lactobacillus*, and *Turicibacter* and significantly negatively correlated with *Bifidobacterium*, *Subdoligranulum*, uncultured_bacterium_f_Lachnospiraceae, and uncultured_bacterium_f_Coriobacteriaceae. Asteraceae was significantly negatively correlated with uncultured_bacterium_f_Lachnospiraceae and *Subdoligranulum*. Amaranthaceae was significantly negatively correlated with *Lactobacillus* but positively correlated with *Akkermansia* and *Subdoligranulum*. Among the animal dietary factors, unclassified_Metazoa was significantly negatively correlated with *Subdoligranulum* and *Bifidobacterium*. *Dasybranchus* was significantly positively correlated with uncultured_bacterium_c_Gammaproteobacteria and either significantly or highly significantly negatively correlated with *Akkermansia*, *Subdoligranulum*, uncultured_bacterium_f_Coriobacteriaceae, *Bifidobacterium*, and uncultured_bacterium_f_Lachnospiraceae.

The CCA and dbRDA results also revealed the influence of food components on the fungal community structures (genus level) in the different sample groups (Figure 5). Plant‐ and animal‐based foods accounted for 34.12% and 34.41% of the total explanation rate for the gut fungal community, respectively. The dbRDA results indicated that plant‐ and animal‐based foods explained 61.21% and 64.10% of the gut fungal communities, respectively. The significant plant and animal dietary factors affecting the fungal community structure in the common crane samples were unclassified_Streptophyta (*R* ^2^ = 0.69, *p* = 0.017) and Unclassified (*R* ^2^ = 0.31, *p* = 0.242), respectively, whereas those affecting the great bustard samples were Poaceae (*R* ^2^ = 0.47, *p* = 0.073) and *Attagenus* (*R* ^2^ = 0.39, *p* = 0.153) (Figure 5G,H,J,K). Again, none of the factors affecting the great bustard samples showed statistical significance. The distinct responses of fungal genera in the different bird samples to dietary factor levels were visualized using Spearman correlation heatmaps (Figure 5I,L). Among the plant dietary factors, Amaranthaceae was highly significantly positively correlated with unidentified fungi and significantly negatively correlated with *Vishniacozyma*. Poaceae was significantly negatively correlated with *Fusicolla* and *Fusarium* and significantly positively correlated with *Meyerozyma* and *Vishniacozyma*. Asteraceae was significantly positively correlated with *Meyerozyma*.

### 3.6 Relationships Between the Gut Microbiota and Metabolites in the Two Bird Species

**FIGURE 6:**
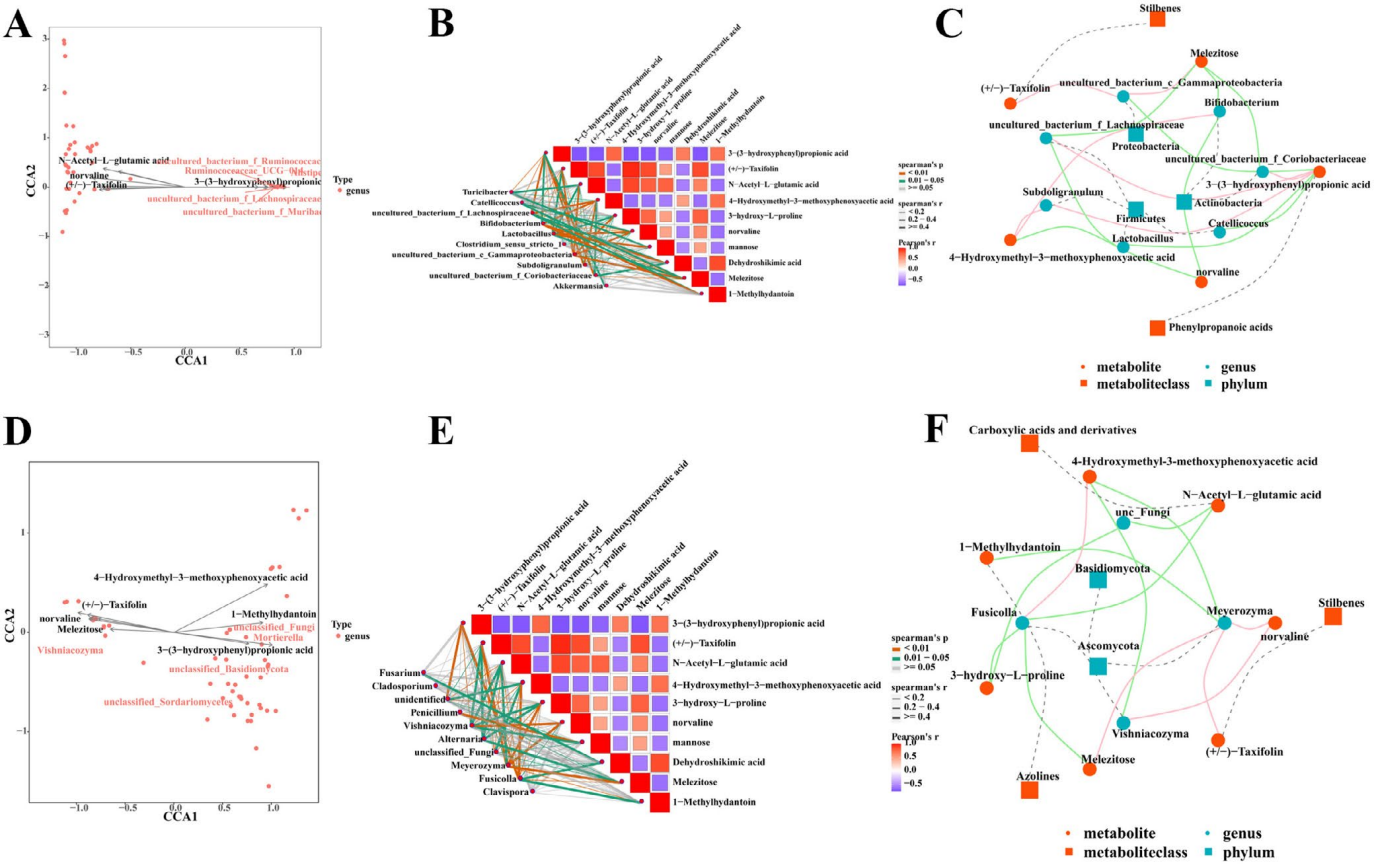
Correlations between the gut microbiota and metabolites in the common crane and great bustard. (A) Canonical correlation analysis (CCA) between bacterial genera and metabolites; (B) Network heatmap of the correlations between the top 10 metabolites and bacterial genera; (C) Advanced network diagram showing the correlations between the gut bacteria and metabolites; (D) CCA between fungal genera and metabolites; (E) Network heatmap of the correlations between the top 10 metabolites and fungal genera; (F) Advanced network diagram showing the correlations between the gut fungi and metabolites.

The metabolite with the greatest influence on the gut bacteria was 3‐(3‐hydroxyphenyl)propionic acid (Figure 6A). It was highly significantly negatively correlated with *Catellicoccus*, uncultured_bacterium_c_Gammaproteobacteria, and *Lactobacillus* and highly significantly positively correlated with uncultured_bacterium_f_Lachnospiraceae, *Subdoligranulum*, and uncultured_bacterium_f_Coriobacteriaceae (|*r*| ≥ 0.8, *p* < 0.01) (Figure 6B,C). Norvaline was highly significantly negatively correlated with uncultured_bacterium_f_Lachnospiraceae and *Bifidobacterium*. Melezitose was highly significantly negatively correlated with uncultured_bacterium_f_Lachnospiraceae, *Bifidobacterium*, and uncultured_bacterium_f_Coriobacteriaceae and highly significantly positively correlated with uncultured_bacterium_c_Gammaproteobacteria. 4‐Hydroxymethyl‐3‐methoxyphenoxyacetic acid was significantly positively correlated with *Bifidobacterium* and *Subdoligranulum* and highly significantly negatively correlated with *Lactobacillus*. (+/−)‐Taxifolin was highly significantly negatively correlated with uncultured_bacterium_c_Gammaproteobacteria.

According to the CCA results of the relationships between the top 10 most abundant fungal genera and top 10 differential metabolites, the metabolite with the greatest influence on the gut fungi was 4‐hydroxymethyl‐3‐methoxyphenoxyacetic acid (Figure 6D). Heatmaps showing the correlations between the fungal genera and metabolites are shown in Figure 6E,F. (+/−)‐Taxifolin was highly significantly positively correlated with *Meyerozyma*. *N*‐Acetyl‐l‐glutamic acid was highly significantly negatively correlated with unclassified_Fungi and *Fusicolla*. 4‐Hydroxymethyl‐3‐methoxyphenoxyacetic acid was highly significantly negatively correlated with *Vishniacozyma* and *Meyerozyma* and highly significantly positively correlated with *Fusicolla*. 3‐Hydroxy‐l‐proline was highly significantly negatively correlated with unclassified_Fungi and *Fusicolla*. Norvaline was highly significantly positively correlated with *Vishniacozyma* and *Meyerozyma*. Melezitose was highly significantly positively correlated with *Meyerozyma*. 1‐Methylhydantoin was highly significantly negatively correlated with *Meyerozyma*.

## 4 Discussion

In China, the great bustard and common crane are ranked as Class I and II protected animals, respectively. To investigate the overwintering conditions of these two rare bird species and their mechanisms of adaptation to the food and environment at this wintering site, we analyzed their diets, gut microbiotas, and fecal metabolites during the overwintering period. Detection of the plant‐ and animal‐based components of fecal samples revealed that the plant‐based diet of the great bustard was mainly composed of species from the families Salicaceae, Rosaceae, Amaranthaceae, Poaceae, and Amaryllidaceae. By contrast, the plant‐based diet of the common crane was primarily composed of Poaceae, which accounted for 84.83% of the total plant components. Previous studies on the winter diet of great bustards in the Tumuji Nature Reserve showed that they mainly consumed plants from the families Fabaceae, Brassicaceae, Asteraceae, and Poaceae (Liu et al. 2018; Bravo et al. 2012; Gooch et al. 2015). These results differ from those of the present study, suggesting that great bustards choose different food sources at various wintering sites to supplement their energy, which suggests an adaptation strategy according to their environment. The food sources of common cranes wintering in the Tianjin Tuanbo Bird Natural Reserve were shown to primarily include *Triticum aestivum* (Poaceae), *Potentilla chinensis* (Rosaceae), *Zea mays* (Poaceae), and *Glycine max* and *Phragmites australis* (Fabaceae) (Wu et al. 2024). In this present study, the primary food source of the common crane was Poaceae, similar to that observed in the Tianjin Tuanbo Bird Natural Reserve study (Liu et al. 2018), indicating stable dietary preferences in this bird species. Animal‐based components were also detected in the feces of both great bustards and common cranes, with the most abundant being species from genus *Rattus*. This indicates that the two bird species may prey on *Rattus* spp. during the wintering period in the Yellow River wetlands. These findings suggest that both bird species adjust their feeding strategies according to the available food sources at their wintering sites to better adapt to their environment.

The compositions of the gut microbiotas of the great bustard and common crane were analyzed using high‐throughput 16S rRNA and ITS sequencing technologies. In the great bustard feces, the dominant bacterial phyla included Firmicutes (62.88%), Actinobacteria (24.89%), Proteobacteria (2.03%), Bacteroidetes (5.93%), and Verrucomicrobia (3.58%). These results are similar to previous findings in great bustards overwintering in Cangzhou (Lu et al. 2021), except that the birds wintering in the Yellow River wetlands of Inner Mongolia have a higher abundance of Verrucomicrobia. By contrast, the dominant bacterial phyla in the common crane were Firmicutes (87.82%) and Proteobacteria (7.83%), which is consistent with a report that Firmicutes, Proteobacteria, and Fusobacteria were the dominant phyla in common cranes overwintering in the Tianjin Tuanbo Bird Natural Reserve (Wu et al. 2024). This suggests that the gut microbiota of the common crane is relatively steady, corresponding to its consistent dietary preferences across different wintering sites. Compared with the common crane, the great bustard exhibited higher gut microbial diversity. Studies have shown that greater dietary diversity correlates with higher gut microbial diversity (Lau et al. 2018). This may explain the differences in gut microbiotas between these two bird species, as the common crane diet primarily consists of Poaceae, whereas the great bustard consumes a wider variety of plant species, leading to higher gut microbial diversity.

The dominant bacterial genera in the great bustard feces included uncultured_bacterium_f_Lachnospiraceae (20.51%), *Bifidobacterium* (18.47%), *Clostridium_sensu_stricto_1* (5.839%), *Subdoligranulum* (4.80%), uncultured_bacterium_f_Coriobacteriaceae (4.56%), and *Akkermansia* (3.58%). Lachnospiraceae, which are core gut bacteria with high hydrolytic enzyme activity (Vacca et al. 2020), are capable of breaking down dietary polysaccharides and cellulose (Wolin et al. 2003) and producing short‐chain fatty acids (SCFAs) as metabolites. SCFAs are beneficial metabolites for the host, given that they enhance intestinal barrier integrity by upregulating tight junctions and mucin production (Brahe et al. 2013), exert anti‐inflammatory effects by inducing regulatory T cells and downregulating proinflammatory cytokines (Portune et al. 2017), and reduce circulating lipid plasma levels and body weight by modulating fatty acid oxidation and synthesis (Esgalhado et al. 2017). *Bifidobacterium* plays significant roles in regulating the host immune system, converting aromatic amino acids into lactate derivatives (Laursen et al. 2021), and antagonizing pathogenic bacteria, thereby maintaining host gut health (Goncharova et al. 1987). *Clostridium_sensu_stricto_1*, which is associated with sphingolipid metabolism, is considered a beneficial microbe against obesity (Cai et al. 2024). *Subdoligranulum* is another SCFA producer (Mahmud et al. 2023), whereas Coriobacteriaceae is linked to longevity and considered a gut probiotic (Liu et al. 2023). *Akkermansia*, a mucosa‐residing gut symbiont (Derrien et al. 2004) with probiotic potential, has been shown to enhance host metabolic functions and immune responses (Collado et al. 2007; Derrien et al. 2008; Derrien et al. 2011; Everard et al. 2013; Zhu et al. 2023) and is surmised to play a critical role in regulating host health under cold stress (Seimbille et al. 2015). These findings suggest that the great bustard harbors a gut microbiota that is rich in probiotics that positively regulate metabolism, indicating that this bird species maintains good health while wintering in the Yellow River wetlands. With regard to the common crane, the dominant bacterial genera were *Turicibacter* (38.67%), *Catellicoccus* (26.89%), *Lactobacillus* (13.71%), and uncultured_bacterium_c_Gammaproteobacteria (4.38%). *Turicibacter* is an SCFA producer (Xu et al. 2022) that positively regulates host metabolic processes, including bile acid and lipid metabolism (Lynch et al. 2023). *Catellicoccus*, a symbiotic bacterium of marine animals and waterbirds (Lawson et al. 2006; Koskey et al. 2014; Safaie et al. 2021), serves as an indicator of fecal contamination in aqueous environments. The bacterium also participates in nutrient transport and bile acid hydrolysis (Góngora et al. 2021). *Lactobacillus* is a probiotic that maintains gut health, antagonizes harmful bacteria, and plays a critical role in host metabolism and wellness. By contrast, Gammaproteobacteria is a common gut pathogen (Huffnagle et al. 2017) that is associated with diseases, and its presence in the gut of the common crane warrants attention.

The analysis of gut fungi showed that the dominant fungal phyla in great bustards and common cranes were Ascomycota (68.74%) and Basidiomycota (20.92%). Similar to gut bacteria, gut fungi are influenced by factors such as the host age, physical condition, and diet and the environment (Su et al. 2022). The predominance of Ascomycota in the gut microbiotas of both bird species aligns with findings from studies on the gut fungi of the North China leopard (Hu et al. 2020) and black‐necked crane (Liu et al. 2020). In the digestive tract, Ascomycota members aid in breaking down complex molecular structures such as lignocellulosic biomass, thereby improving the digestibility of animal feed (Mangiola et al. 2016; Gomez et al. 2017). Our results indicate that the Ascomycota play a role in helping great bustards and common cranes digest their primarily plant‐based diets during winter, aiding in energy storage. At the genus level, the most abundant fungal genera were *Fusarium* (8.10%) and *Cladosporium* (6.25%), both of which are opportunistic pathogens (Batra et al. 2019) commonly found in soil and on plants. Taken together, these findings indicate that gut fungi are influenced by the environment.

The analysis of fecal samples revealed significant differences in gut metabolites between the two bird species. The highly differential metabolites were primarily enriched in metabolic pathways related to plant‐based food metabolism, including plant hormone signal transduction, galactose metabolism, ubiquinone and other terpenoid‐quinone biosynthesis, bisphenol degradation, folate biosynthesis, flavonoid biosynthesis, fructose and mannose metabolism, biosynthesis of various secondary metabolites—part 2, and biosynthesis of plant hormones. These results further confirm that both great bustards and common cranes rely primarily on plant‐based food during the wintering period in the Yellow River wetlands of Inner Mongolia.

The correlation analysis between dietary factors and the gut microbiotas showed that the diversity of gut bacteria and fungi was significantly influenced by plant‐based, but not animal‐based, foods. This finding further supports the conclusion that great bustards and common cranes mainly consume plant‐based foods during winter, which is consistent with the findings from previous studies (Bravo et al. 2012; Gooch et al. 2015). Additionally, plant‐based dietary factors significantly influenced the gut microbial communities of the common crane but had no significant effect on those of the great bustard. Among the plant‐based dietary factors, Poaceae displayed a significant positive correlation with the gut bacteria of the common crane but showed a significant negative correlation with gut bacteria and fungi of the great bustard. Amaranthaceae was negatively correlated with the gut bacteria of the common crane but positively correlated with those of the great bustard. This is likely because the common crane primarily feeds on Poaceae, and its gut microbiota is remodeled to digest and metabolize this ingested food, thereby providing energy for metabolism (Zhang et al. 2024). By contrast, the diet of the great bustard is more diverse, and no single plant‐based food component drives the composition of its gut microbiota. Instead, the microbiota is shaped by a combination of dietary factors. Therefore, this study indicates that diet is a key factor driving gut microbe communities (Ley et al. 2006).

The correlation analysis between the gut metabolites and microbiota showed that 3‐(3‐hydroxyphenyl)propionic acid, a microbial‐generated flavonoid, had the most significant impact on the microbial community (Feng et al. 2022). This metabolite was significantly upregulated in the great bustard, the diet of which includes plants from the families Salicaceae, Rosaceae, Amaranthaceae, Poaceae, and Amaryllidaceae. In particular, Rosaceae plants produce secondary flavonoid metabolites (Jakimiuk and Tomczyk 2024). These results suggest that the overwintering great bustards consume Rosaceae plants, which are hydrolyzed and metabolized by gut microbes with hydrolase functions, such as members of the Lachnospiraceae (Vacca et al. 2020), resulting in flavonoid metabolites. These metabolites exhibit antioxidative and anti‐inflammatory activities (Majee et al. 2023) and play essential roles in maintaining gut homeostasis and host health (Serafini et al. 2010).

The metabolite with the most significant effect on gut fungi was 4‐hydroxymethyl‐3‐methoxyphenoxyacetic acid, which was significantly upregulated in the great bustard. This phenolic acid is derived from the breakdown of lignin and cellulose in woody plants such as trees and shrubs (Singh et al. 2023) or Poaceae plants (Xu et al. 2017). Unlike common cranes that primarily consume Poaceae, great bustards consume a smaller proportion of Poaceae but also include some Rosaceae and predominantly woody plants in their diet. Although both bird species produce phenolic acid metabolites from plant‐based foods, the levels of 4‐hydroxymethyl‐3‐methoxyphenoxyacetic acid were higher in the great bustards. This may be due to the differences in gut bacteria and fungi between the two bird species, resulting in distinct metabolic pathways for digesting plant‐based foods and producing different metabolites. The correlation analysis showed that 4‐hydroxymethyl‐3‐methoxyphenoxyacetic acid was significantly negatively correlated with the abundance of *Vishniacozyma* and *Meyerozyma* in the gut of the common crane but significantly positively correlated with the abundance of *Fusicolla* in the gut of the great bustard. This suggests that great bustards rely primarily on *Fusicolla* to metabolize plant‐based foods and produce this metabolite. Phenolic acids were previously shown to increase the abundance of *Fusicolla* in soil, indicating a positive correlation between this fungal genus and phenolic acid metabolites, which is consistent with the trend observed in our present study (Clocchiatti et al. 2021). In the gut of the common crane, this metabolite was produced in smaller amounts, possibly because *Vishniacozyma* and *Meyerozyma* metabolize plant‐based foods through other pathways, resulting in different metabolites. These results suggest that even when the same plant‐based food (e.g., Poaceae) is consumed, it may be metabolized by different gut microorganisms in the two bird species, resulting in different metabolites being produced. The differences in the gut microbiotas of the two bird species are shaped by their dietary diversity. Thus, different dietary choices remodel the bacterial and fungal communities in the gut, enabling the digestion and metabolism of plant‐based foods through different pathways, which leads to the production of distinct metabolites that perform different functions in the host.

This study had several limitations. First, the sample size was relatively small owing to the inherent difficulty in obtaining samples from rare and protected wildlife. The small sample size may have led to high inter‐individual variation in diet composition and limitations in the statistical analysis of the data. Nevertheless, a high variability in diet composition among individuals may also stem from genuine biological phenomena in wild birds, such as individual foraging specialization (Woo et al. 2008). In omnivorous or generalist bird species, different individuals commonly utilize different food patches, resulting in high variation in diet composition. This variation does not indicate that the data are unreliable; instead, it provides valuable ecological insights into the dietary plasticity and adaptive strategies of the species during the wintering period. With regard to the statistical analysis, using uncorrected statistics to determine the significance of correlation analyses implies that some observed associations may be uncertain. Therefore, our conclusions regarding diet–microbiota–metabolite interactions should be further investigated in future studies with larger sample sizes and additional experimental validation. Furthermore, the use of DNA metabarcoding to analyze diet compositions has certain limitations, including potential PCR amplification bias, differences in DNA degradation rates among food items during digestion, incomplete reference databases, and the challenge of accurately quantifying actual food intake from sequence reads. In future studies, DNA metabarcoding should be combined with fecal microhistological analysis (Liu et al. 2022) to more accurately reflect the diet composition of wildlife.

In summary, the diets and gut microbiotas of the great bustard and common crane during winter in the Yellow River wetlands of Inner Mongolia were investigated using high‐throughput sequencing technology. The results showed that both bird species mainly consumed plant‐based foods. The common crane primarily fed on Poaceae, whereas the great bustard consumed a more complex diet that included plants from the Salicaceae, Rosaceae, Amaranthaceae, Poaceae, and Amaryllidaceae families. The different diets drove the diversity and composition of the gut microbiotas, with the great bustard exhibiting higher gut microbial diversity. The gut microbiota and metabolites of the common crane were primarily influenced by Poaceae, whereas those of the great bustard were influenced by multiple factors. Moreover, the great bustard harbored more probiotic bacteria and beneficial metabolites (e.g., flavonoids and phenolic acids) that are advantageous for host health, whereas the common cranes harbored more probiotic fungi. The findings from this study indirectly indicate that great bustards and common cranes are well adapted to the environment of the Yellow River wetlands during the wintering period. Therefore, these wetlands provide a favorable habitat for these two rare bird species.

## Author Contributions

**Li Gao:** conceptualization (equal), formal analysis (equal), methodology (equal), writing – original draft (equal), writing – review and editing (equal). **Haitao Fang:** conceptualization (equal), investigation (equal), visualization (equal). **Hongda Pei:** data curation (equal), project administration (equal), software (equal), visualization (equal). **Junlan Li:** formal analysis (equal), writing – original draft (equal), writing – review and editing (equal). **Li Liu:** conceptualization (equal), writing – review and editing (equal).

## Funding

This work was supported by the Study on Habitat Suitability of Great Bustard Based on Maximum Entropy Model (01108023/005), the Basic Investigation Project of Ministry of Science and Technology—Animal Resources, and Investigation in Desert and Semi desert Areas of the Inner Mongolian Plateau (2023FY100300).

## Ethics Statement

All handling and study procedures were performed in accordance with the recommendations for animal care and ethics of China. Noninvasive techniques were used to collect fecal samples. The Animal Ethics and Welfare Committee of Baotou Teachers College approved this project (AEWC‐BTTC2021005).

## Conflicts of Interest

The authors declare no conflicts of interest.

## Data Availability

Raw sequence reads are deposited in the SRA (BioProject PRJNA1201601 (Bacteria), BioProject PRJNA1201602 (Fungi), BioProject PRJNA1202360 (Plant‐diet), and BioProject PRJNA1202364 (Animal‐diet)). The names of the repository/repositories and accession number(s) can be found below: http://www.ncbi.nlm.nih.gov/bioproject/PRJNA1201601, http://www.ncbi.nlm.nih.gov/bioproject/PRJNA1201602, http://www.ncbi.nlm.nih.gov/bioproject/PRJNA1202360, and http://www.ncbi.nlm.nih.gov/bioproject/PRJNA1202364.

## References

[ece373814-bib-0001] Batra, N. , H. Kaur , S. Mohindra , S. Singh , A. S. Shamanth , and S. M. Rudramurthy . 2019. “Cladosporium Sphaerospermum Causing Brain Abscess, a Saprophyte Turning Pathogen: Case and Review of Published Reports.” Journal of Medical Mycology 29, no. 2: 180–184. 10.1016/j.mycmed.2019.04.005. 31056403

[ece373814-bib-0002] Brahe, L. K. , A. Astrup , and L. H. Larsen . 2013. “Is Butyrate the Link Between Diet, Intestinal Microbiota and Obesity‐Related Metabolic Diseases?” Obesity Reviews 14, no. 12: 950–959. 10.1111/obr.12068. 23947604

[ece373814-bib-0003] Bravo, C. , C. Ponce , C. Palacín , and J. Carlos Alonso . 2012. “Diet of Young Great Bustards *Otis tarda* in Spain: Sexual and Seasonal Differences.” Bird Study 59: 243–251. 10.1080/00063657.2012.662940.

[ece373814-bib-0004] Cai, K. , X. Y. Cao , F. Chen , et al. 2024. “Xianlian Jiedu Decoction Alleviates Colorectal Cancer by Regulating Metabolic Profiles, Intestinal Microbiota and Metabolites.” Phytomedicine 128: 155385. 10.1016/j.phymed.2024.155385. 38569292

[ece373814-bib-0005] Clocchiatti, A. , S. E. Hannula , M. van den Berg , M. P. J. Hundscheid , and W. de Boer . 2021. “Evaluation of Phenolic Root Exudates as Stimulants of Saprotrophic Fungi in the Rhizosphere.” Frontiers in Microbiology 12: 644046. 10.3389/fmicb.2021.644046. 33936001 PMC8079663

[ece373814-bib-0006] Collado, M. C. , M. Derrien , E. Isolauri , W. M. De Vos , and S. Salminen . 2007. “Intestinal Integrity and *Akkermansia muciniphila* , a Mucin‐Degrading Member of the Intestinal Microbiota Present in Infants, Adults, and the Elderly.” Applied and Environmental Microbiology 73, no. 23: 7767–7770. 10.1128/AEM.01477-07. 17933936 PMC2168041

[ece373814-bib-0007] Derrien, M. , M. C. Collado , K. Ben‐Amor , S. Salminen , and W. M. de Vos . 2008. “The Mucin Degrader *Akkermansia muciniphila* Is an Abundant Resident of the Human Intestinal Tract.” Applied and Environmental Microbiology 74, no. 5: 1646–1648. 10.1128/AEM.01226-07. 18083887 PMC2258631

[ece373814-bib-0008] Derrien, M. , P. Van Baarlen , G. Hooiveld , et al. 2011. “Modulation of Mucosal Immune Response, Tolerance, and Proliferation in Mice Colonized by the Mucin‐Degrader *Akkermansia muciniphila* .” Frontiers in Microbiology 2: 166. 10.3389/fmicb.2011.00166. 21904534 PMC3153965

[ece373814-bib-0009] Derrien, M. , E. E. Vaughan , C. M. Plugge , and W. M. de Vos . 2004. “ *Akkermansia muciniphila* gen. nov., sp. nov., a Human Intestinal Mucin‐Degrading Bacterium.” International Journal of Systematic and Evolutionary Microbiology 54, no. Pt 5: 1469–1476. 10.1099/ijs.0.02873-0. 15388697

[ece373814-bib-0010] Esgalhado, M. , J. A. Kemp , N. R. Damasceno , D. Fouque , and D. Mafra . 2017. “Short‐Chain Fatty Acids: A Link Between Prebiotics and Microbiota in Chronic Kidney Disease.” Future Microbiology 12: 1413–1425. 10.2217/fmb-2017-0059. 29027814

[ece373814-bib-0011] Everard, A. , C. Belzer , L. Geurts , et al. 2013. “Cross‐Talk Between *Akkermansia muciniphila* and Intestinal Epithelium Controls Diet‐Induced Obesity.” Proceedings of the National Academy of Sciences of the United States of America 110, no. 22: 9066–9071. 10.1073/pnas.1219451110. 23671105 PMC3670398

[ece373814-bib-0012] Feng, J. , C. Ge , W. Li , and R. Li . 2022. “3‐(3‐Hydroxyphenyl)propionic Acid, a Microbial Metabolite of Quercetin, Inhibits Monocyte Binding to Endothelial Cells via Modulating E‐Selectin Expression.” Fitoterapia 156: 105071. 10.1016/j.fitote.2021.105071. 34743931

[ece373814-bib-0013] Gao, L. , Y. Ma , L. Wang , et al. 2025. “Combined Metabolome and Transcriptome Analysis Revealed That MSTN Regulated the Process of Bovine Fatty Acid Metabolism in Gut.” Frontiers in Veterinary Science 12: 1541257. 10.3389/fvets.2025.1541257. 40357198 PMC12066744

[ece373814-bib-0014] Gomez, D. E. , L. G. Arroyo , M. C. Costa , L. Viel , and J. S. Weese . 2017. “Characterization of the Fecal Bacterial Microbiota of Healthy and Diarrheic Dairy Calves.” Journal of Veterinary Internal Medicine 31, no. 3: 928–939. 10.1111/jvim.14695. 28390070 PMC5435056

[ece373814-bib-0015] Goncharova, G. I. , L. P. Semenova , A. M. Liannaia , et al. 1987. “Human Bifidobacterium Flora, Its Normalizing and Protective Functions.” Antibiotiki i Meditsinskaya Biotekhnologiya 32, no. 3: 179–183 (in Russian). 3555325

[ece373814-bib-0016] Góngora, E. , K. H. Elliott , and L. Whyte . 2021. “Gut Microbiome Is Affected by Inter‐Sexual and Inter‐Seasonal Variation in Diet for Thick‐Billed Murres ( *Uria lomvia* ).” Scientific Reports 11, no. 1: 1200. 10.1038/s41598-020-80557-x. 33441848 PMC7806582

[ece373814-bib-0017] Gooch, S. , K. Ashbrook , A. Taylor , and T. Székely . 2015. “Using Dietary Analysis and Habitat Selection to Inform Conservation Management of Reintroduced Great Bustards *Otis tarda* in an Agricultural Landscape.” Bird Study 62: 289–302. 10.1080/00063657.2015.105099.

[ece373814-bib-0018] Grond, K. , and A. Jumpponen . 2018. “The Avian Gut Microbiota: Community, Physiology and Function in Wild Birds.” Journal of Avian Biology 49: e01788. 10.1111/jav.01788.

[ece373814-bib-0019] Hu, Y. , P. F. Lang , H. T. Wang , et al. 2020. “Analysis of Intestinal Fungus Diversity of Wild and Captive North‐Chinese Leopard ( *Panthera pardus japonensis* ) Based on High‐Throughput Sequencing.” Chinese Journal of Wildlife 41, no. 1: 5–14 (in Chinese). 10.19711/j.cnki.issn2310-1490.2020.01.002.

[ece373814-bib-0020] Huffnagle, G. B. , R. P. Dickson , and N. W. Lukacs . 2017. “The Respiratory Tract Microbiome and Lung Inflammation: A Two‐Way Street.” Mucosal Immunology 10, no. 2: 299–306. 10.1038/mi.2016.108. 27966551 PMC5765541

[ece373814-bib-0021] Jakimiuk, K. , and M. Tomczyk . 2024. “A Review of the Traditional Uses, Phytochemistry, Pharmacology, and Clinical Evidence for the Use of the Genus Alchemilla (Rosaceae).” Journal of Ethnopharmacology 320: 117439. 10.1016/j.jep.2023.117439. 37981119

[ece373814-bib-0022] Kessler, A. E. , N. Batbayar , T. Natsagdorj , D. Batsuur’ , and A. T. Smith . 2013. “Satellite Telemetry Reveals Long‐Distance Migration in the Asian Great Bustard *Otis tarda dybowskii* .” Journal of Avian Biology 44: 311–320. 10.1111/j.1600-048X.2013.00072.x.

[ece373814-bib-0023] Koskey, A. M. , J. C. Fisher , M. F. Traudt , R. J. Newton , and S. L. McLellan . 2014. “Analysis of the Gull Fecal Microbial Community Reveals the Dominance of *Catellicoccus marimammalium* in Relation to Culturable Enterococci.” Applied and Environmental Microbiology 80, no. 2: 757–765. 10.1128/AEM.02414-13. 24242244 PMC3911088

[ece373814-bib-0024] Lau, S. K. P. , J. L. L. Teng , T. H. Chiu , et al. 2018. “Differential Microbial Communities of Omnivorous and Herbivorous Cattle in Southern China.” Computational and Structural Biotechnology Journal 16: 54–60. 10.1016/j.csbj.2018.02.004. 29686799 PMC5910514

[ece373814-bib-0025] Laursen, M. F. , M. Sakanaka , N. von Burg , et al. 2021. “Bifidobacterium Species Associated With Breastfeeding Produce Aromatic Lactic Acids in the Infant Gut.” Nature Microbiology 6, no. 11: 1367–1382. 10.1038/s41564-021-00970-4. PMC8556157 34675385

[ece373814-bib-0026] Lawson, P. A. , M. D. Collins , E. Falsen , and G. Foster . 2006. “ *Catellicoccus marimammalium* gen. nov., sp. nov., a Novel Gram‐Positive, Catalase‐Negative, Coccus‐Shaped Bacterium From Porpoise and Grey Seal.” International Journal of Systematic and Evolutionary Microbiology 56, no. Pt 2: 429–432. 10.1099/ijs.0.63874-0. 16449452

[ece373814-bib-0027] Ley, R. E. , P. J. Turnbaugh , S. Klein , and J. I. Gordon . 2006. “Microbial Ecology: Human Gut Microbes Associated With Obesity.” Nature 444, no. 7122: 1022–1023. 10.1038/4441022a. 17183309

[ece373814-bib-0028] Liao, B. , T. Feng , S. Hou , H. Liu , and J. Feng . 2025. “Simulated Microgravity Confines and Fragments the Straw‐Based Lignocellulose Degrading Microbial Community.” Microbiology Spectrum 13, no. 6: e0246624. 10.1128/spectrum.02466-24. 40237517 PMC12131793

[ece373814-bib-0029] Liu, G. , X. Y. Lv , M. M. Zhang , et al. 2020. “The Intestinal Fungal Diversity of Wintering Black‐Necked Cranes ( *Grus nigricollis* ) at Caohai, Guizhou.” Heilongjiang Animal Science and Veterinary Medicine 5: 128–132+152‐153 (in Chinese). 10.13881/j.cnki.hljxmsy.2019.02.0084.

[ece373814-bib-0030] Liu, G. , A. B. A. Shafer , X. Hu , et al. 2018. “Meta‐Barcoding Insights Into the Spatial and Temporal Dietary Patterns of the Threatened Asian Great Bustard ( *Otis tarda dybowskii* ) With Potential Implications for Diverging Migratory Strategies.” Ecology and Evolution 8, no. 3: 1736–1745. 10.1002/ece3.3791. 29435248 PMC5792609

[ece373814-bib-0031] Liu, L. , X. Liu , C. Du , et al. 2022. “Spring Diet and Energy Intake of Whooper Swans ( *Cygnus cygnus* ) at the Yellow River National Wetland in Baotou, China.” PLoS One 17, no. 2: e0264528. 10.1371/journal.pone.0264528. 35226691 PMC8884505

[ece373814-bib-0032] Liu, L. , X. G. Liu , Y. Sun , et al. 2019. “Trace Elements in the Feathers of Waterfowl From Nanhaizi Wetland, Baotou, China.” Bulletin of Environmental Contamination and Toxicology 102, no. 6: 778–783. 10.1007/s00128-019-02596-z. 30918995

[ece373814-bib-0033] Liu, X. , L. Zou , C. Nie , et al. 2023. “Mendelian Randomization Analyses Reveal Causal Relationships Between the Human Microbiome and Longevity.” Scientific Reports 13, no. 1: 5127. 10.1038/s41598-023-31115-8. 36991009 PMC10052271

[ece373814-bib-0034] Lu, Z. , S. Li , H. Li , Z. Wang , D. Meng , and J. Liu . 2021. “The Gut Microbiota of Wild Wintering Great Bustard ( *Otis tarda dybowskii* ): Survey Data From Two Consecutive Years.” PeerJ 9: e12562. 10.7717/peerj.12562. 34909281 PMC8641483

[ece373814-bib-0035] Lynch, J. B. , E. L. Gonzalez , K. Choy , et al. 2023. “Gut Microbiota Turicibacter Strains Differentially Modify Bile Acids and Host Lipids.” Nature Communications 14, no. 1: 3669. 10.1038/s41467-023-39403-7. PMC10281990 37339963

[ece373814-bib-0036] Mahmud, M. R. , C. Jian , M. K. Uddin , et al. 2023. “Impact of Intestinal Microbiota on Growth Performance of Suckling and Weaned Piglets.” Microbiology Spectrum 11, no. 3: e0374422. 10.1128/spectrum.03744-22. 37022154 PMC10269657

[ece373814-bib-0037] Majee, C. , R. Mazumder , A. N. Choudhary , and Salahuddin . 2023. “An Insight Into the Hepatoprotective Activity and Structure‐Activity Relationships of Flavonoids.” Mini‐Reviews in Medicinal Chemistry 23, no. 2: 131–149. 10.2174/1389557522666220602141142. 35657045

[ece373814-bib-0038] Mangiola, F. , G. Ianiro , F. Franceschi , S. Fagiuoli , G. Gasbarrini , and A. Gasbarrini . 2016. “Gut Microbiota in Autism and Mood Disorders.” World Journal of Gastroenterology 22, no. 1: 361–368. 10.3748/wjg.v22.i1.361. 26755882 PMC4698498

[ece373814-bib-0039] Martín, C. A. , J. C. Alonso , J. A. Alonso , et al. 2007. “Sex‐Biased Juvenile Survival in a Bird With Extreme Size Dimorphism, the Great Bustard *Otis tarda* .” Journal of Avian Biology 38: 335–346. 10.1111/j.2007.0908-8857.03811.x.

[ece373814-bib-0040] Pereira, H. , N. Chakarov , B. A. Caspers , et al. 2024. “The Gut Microbiota of Three Avian Species Living in Sympatry.” BMC Ecology and Evolution 24, no. 1: 144. 10.1186/s12862-024-02329-9. 39574002 PMC11580620

[ece373814-bib-0041] Portune, K. J. , A. Benítez‐Páez , E. M. Del Pulgar , et al. 2017. “Gut Microbiota, Diet, and Obesity‐Related Disorders—The Good, the Bad, and the Future Challenges.” Molecular Nutrition & Food Research 61, no. 1. 10.1002/mnfr.201600252. 27287778

[ece373814-bib-0042] Safaie, A. , C. J. Weiskerger , M. B. Nevers , M. N. Byappanahalli , and M. S. Phanikumar . 2021. “Evaluating the Impacts of Foreshore Sand and Birds on Microbiological Contamination at a Freshwater Beach.” Water Research 190: 116671. 10.1016/j.watres.2020.116671. 33302038

[ece373814-bib-0043] Seimbille, Y. , N. Zamboni , S. Hapfelmeier , et al. 2015. “Gut Microbiota Orchestrates Energy Homeostasis During Cold.” Cell 163, no. 6: 1360–1374. 10.1016/j.cell.2015.11.004. 26638070

[ece373814-bib-0044] Serafini, M. , I. Peluso , and A. Raguzzini . 2010. “Flavonoids as Anti‐Inflammatory Agents.” Proceedings of the Nutrition Society 69, no. 3: 273–278. 10.1017/S002966511000162X. 20569521

[ece373814-bib-0045] Singh, A. K. , R. K. Singla , and A. K. Pandey . 2023. “Chlorogenic Acid: A Dietary Phenolic Acid With Promising Pharmacotherapeutic Potential.” Current Medicinal Chemistry 30, no. 34: 3905–3926. 10.2174/0929867329666220816154634. 35975861

[ece373814-bib-0046] Su, R. N. , E. Myagmarsuren , D. Menggen , et al. 2022. “Fungal Diversity of Siberian Musk Deer and Forest Musk Deer Feces in Winter and Summer.” Mycosystema 41, no. 1: 17–29 (in Chinese). 10.13346/j.mycosystema.210117.

[ece373814-bib-0047] Vacca, M. , G. Celano , F. M. Calabrese , P. Portincasa , M. Gobbetti , and M. de Angelis . 2020. “The Controversial Role of Human Gut Lachnospiraceae.” Microorganisms 8, no. 4: 573. 10.3390/microorganisms8040573. 32326636 PMC7232163

[ece373814-bib-0048] Wang, S. , C. Liu , Z. Zhang , et al. 2026. “Ecological and Evolutionary Drivers of Trait‐Based Symbiosis and Phylosymbiosis in Avian Gut Microbiota.” Science China Life Sciences. 10.1007/s11427-025-3197-2. 41575703

[ece373814-bib-0049] Wolin, M. J. , T. L. Miller , M. D. Collins , and P. A. Lawson . 2003. “Formate‐Dependent Growth and Homoacetogenic Fermentation by a Bacterium From Human Feces: Description of *Bryantella formatexigens* Gen. Nov., sp. Nov.” Applied and Environmental Microbiology 69, no. 10: 6321–6326. 10.1128/AEM.69.10.6321-6326.2003. 14532100 PMC201199

[ece373814-bib-0050] Woo, K. J. , K. H. Elliott , M. Davidson , A. J. Gaston , and G. K. Davoren . 2008. “Individual Specialization in Diet by a Generalist Marine Predator Reflects Specialization in Foraging Behaviour.” Journal of Animal Ecology 77, no. 6: 1082–1091. 10.1111/j.1365-2656.2008.01429.x. 18624834

[ece373814-bib-0051] Wu, H. , N. Wu , X. Liu , L. Zhang , and D. Zhao . 2024. “Diet Drives Gut Bacterial Diversity of Wild and Semi‐Captive Common Cranes ( *Grus grus* ).” Animals 14, no. 11: 1566. 10.3390/ani14111566. 38891613 PMC11171321

[ece373814-bib-0052] Xiao, K. , Y. Fan , Z. Zhang , et al. 2021. “Covariation of the Fecal Microbiome With Diet in Nonpasserine Birds.” mSphere 6, no. 3: e00308‐21. 10.1128/mSphere.00308-21. 33980682 PMC8125056

[ece373814-bib-0053] Xu, L. , P. Wang , B. Ali , et al. 2017. “Changes of the Phenolic Compounds and Antioxidant Activities in Germinated Adlay Seeds.” Journal of the Science of Food and Agriculture 97, no. 12: 4227–4234. 10.1002/jsfa.8298. 28251647

[ece373814-bib-0054] Xu, Y. , Y. Yang , B. Li , Y. Xie , Y. Shi , and G. le . 2022. “Dietary Methionine Restriction Improves Gut Microbiota Composition and Prevents Cognitive Impairment in D‐Galactose‐Induced Aging Mice.” Food & Function 13, no. 24: 12896–12914. 10.1039/d2fo03366f. 36444912

[ece373814-bib-0055] Zhang, S. , C. Zhou , Z. Dong , et al. 2024. “The Diet‐Intestinal Microbiota Dynamics and Adaptation in an Elevational Migration Bird, the Himalayan Bluetail ( *Tarsiger rufilatus* ).” Ecology and Evolution 14, no. 7: e11617. 10.1002/ece3.11617. 38952660 PMC11214064

[ece373814-bib-0056] Zhu, P. , and L. Xu . 2025. “Friends or Foes? The Indispensable Role of Gut Microbiota in Plant–Fungus–Herbivore Interactions.” Integrative Zoology 20, no. 5: 1093–1095. 10.1111/1749-4877.13019. 40673794

[ece373814-bib-0057] Zhu, Z. , J. Cai , W. Hou , et al. 2023. “Microbiome and Spatially Resolved Metabolomics Analysis Reveal the Anticancer Role of Gut *Akkermansia muciniphila* by Crosstalk With Intratumoral Microbiota and Reprogramming Tumoral Metabolism in Mice.” Gut Microbes 15, no. 1: 2166700. 10.1080/19490976.2023.2166700. 36740846 PMC9904296

